# Septic Arthritis of the Temporomandibular Joint in a Geriatric Patient: A Case Report

**DOI:** 10.7759/cureus.73026

**Published:** 2024-11-04

**Authors:** Vural Akın, Hasan Yasan, Deniz Karaosmanoğlu Akın, Çaglar Irmak, Erdinç Tanlak

**Affiliations:** 1 Department of Otorhinolaryngology and Head and Neck Surgery, Yüksekova State Hospital, Hakkari, TUR; 2 Department of Otorhinolaryngology and Head and Neck Surgery, Süleyman Demirel University, Isparta, TUR; 3 Department of Endodontics, Hakkari Oral and Dental Health Center, Hakkari, TUR; 4 Department of Infectious Diseases and Clinical Microbiology, Yüksekova State Hospital, Hakkari, TUR; 5 Department of Radiology, Yüksekova State Hospital, Hakkari, TUR

**Keywords:** arthritis, geriatrics, infectious arthritis, temporomandibular joint, trismus

## Abstract

Septic arthritis of the temporomandibular joint is a rare pathology. There is no consensus in the literature regarding etiopathogenesis and treatment. Pain, swelling, trismus, and mandibular deviation are common. If diagnosis is delayed and appropriate treatment is not given, complications such as osteomyelitis, ankylosis, and systemic spread may develop. This article presents the case of an 85-year-old male patient with septic arthritis of the temporomandibular joint, including clinical, laboratory, and radiological features, accompanied by relevant literature. Since treatment was started early and there were no comorbidities, the disease was controlled without the need for surgical intervention or any complications. The case presented in this article is one of the oldest patients in the literature. Decreased oral intake due to loss of function of the temporomandibular joint in geriatric patients may predispose them to systemic spread.

## Introduction

Septic arthritis of the temporomandibular joint (SATMJ) is a rare pathology [1]. Its rarity leads to a lack of consensus about the etiopathogenesis and management of the disease. SATMJ should be suspected in patients presenting with complaints such as preauricular pain, swelling, trismus, and mandibular deviation. SATMJ can affect the bone, cartilage, joint capsule, and adjacent muscles. Although it is often an acute condition, it can become chronic [2,3]. It usually occurs following infective conditions such as oropharyngeal infections, ear infections, and dental infections [1,2,4]. It is important for clinicians to recognize this pathology as it may lead to complications such as osteomyelitis, ankylosis, and systemic spread if the diagnosis is delayed and appropriate treatment is not given [1,2]. In light of the literature, this article presents the clinical, laboratory, and radiological features of SATMJ detected in an 85-year-old male patient. Informed consent was obtained from the patient for this report.

## Case presentation

**Figure 1:**
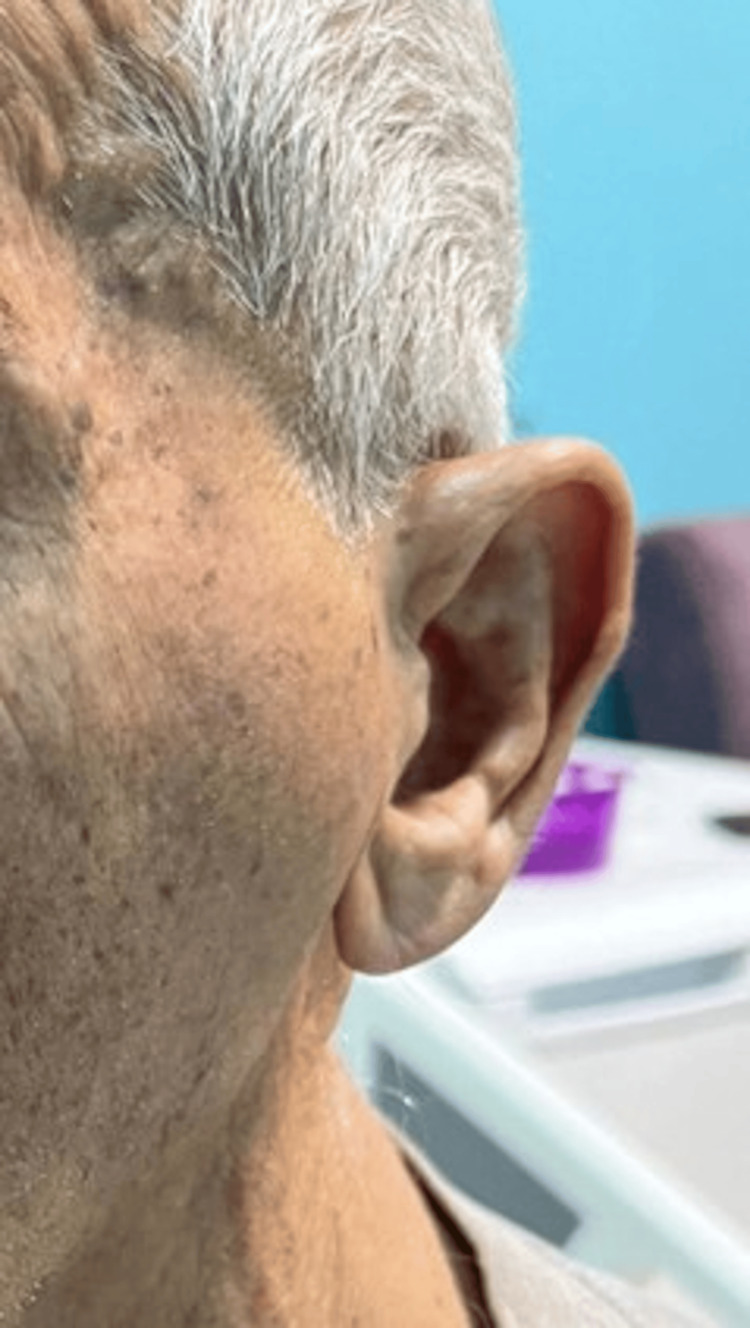
Image of the swelling in the left temporomandibular joint upon initial evaluation

An 85-year-old male patient presented with complaints of swelling and pain in front of his left ear for three days. He said that the pain intensified upon opening his mouth. There was no fever. There was no recent history of infection in the head and neck area. Oral intake decreased due to increased pain with jaw movements. The patient had no known systemic disease and was not using any medication. On physical examination, there was swelling in the left temporomandibular joint (TMJ) region (Figure 1).

This area was slightly hyperemic. Palpation of this region was painful, and there was a localized temperature increase. The fluctuant area was not palpated. Jaw movements were symmetrical, but the patient felt pain when opening his mouth. Mouth opening was minimally restricted due to pain. There were no infective findings in the oral cavity, oropharynx, ear, nose, and neck examination. There was no active pathology during the patient's dental examination. The results of laboratory investigations are presented in Table 1.

**Table 1:** Laboratory investigations CRP: C-reactive protein

Test	Observed Value	Reference Range
Leukocyte	6.2×10^3^ µ/L	3.5-9.5×10^3^ µ/L
Neutrophil	3.63×10^3^ µ/L	1.8-6.3×10^3^ µ/L
Lymphocyte	1.76×10^3^ µ/L	1.1-3.2×10^3^ µ/L
Monocyte	0.61×10^3^ µ/L	0.1-0.6×10^3^ µ/L
CRP	74.6 mg/L	0–5 mg/L

**Figure 2:**
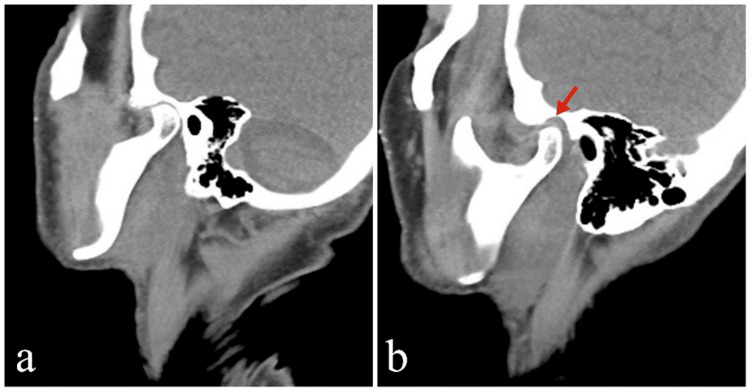
Sagittal cross-sectional images of neck computed tomography Compared to the right temporomandibular joint (a), the left temporomandibular joint (b) exhibits a widening of the joint space and an appearance compatible with inflammation in the adjacent soft tissues (red arrow). No pathology was observed in the bone tissue.

**Figure 3:**
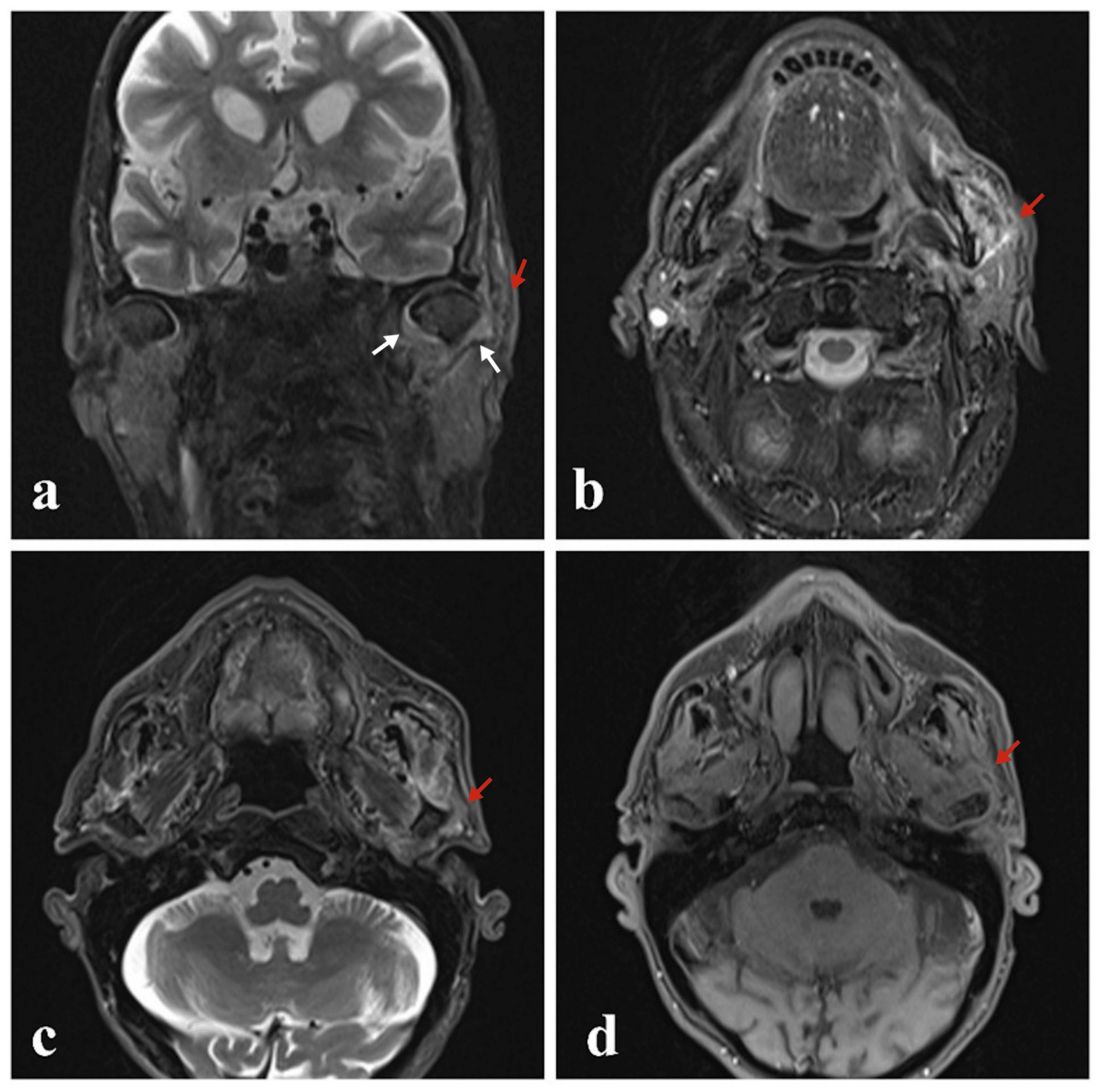
Images of facial magnetic resonance imaging Edema and increased thickness were observed in the masticatory muscles, especially the masseter muscle, in the coronal T2 FS (a), axial T2 FS (b,c), and axial T1 FS (d) images (red arrows). There was a minimal fluid increase in the left temporomandibular joint in the coronal T2 FS (a) images (white arrows).

Figure 2 presents findings on neck computed tomography (CT) scan, and Figure 3 presents findings on facial magnetic resonance imaging (MRI).

Based on the history, physical examination, laboratory tests, and imaging studies, the patient was diagnosed with SATMJ and was hospitalized for antibiotic therapy and follow-up. No interventional procedure was performed because no image was consistent with destruction or abscess in the joint. The Infectious Diseases Department was consulted, and ampicillin-sulbactam at a dose of 4×1.5 g and 1×750 mg levofloxacin IV were administered empirically for three days.

The patient's clinic improved significantly, and the swelling and pain decreased. The control C-reactive protein (CRP) value was 34.2 mg/L. Following a positive treatment response and the normalization of the patient's oral intake, the Infectious Diseases Department was consulted, and the patient was discharged with a prescription for 2×1000 mg amoxicillin-clavulanate and 1×750 mg levofloxacin per oral. Antibiotic therapy was completed in six weeks. The patient visited the outpatient clinic for weekly checkups. At the end of the sixth week, the CRP value returned to normal. There was no evidence of recurrence or sequelae at the third-month follow-up.

## Discussion

Omiunu et al. identified 93 cases reported between 1969 and 2020 in their review. The average age of the patients was 35.7 years, and 60.2% of the patients were male. They reported seven patients over the age of 65, and the oldest case was 85 years [1]. According to these results, SATMJ is relatively rare in the geriatric population. Pathogenic microorganisms that cause SATMJ spread to the TMJ mostly hematogenously or from the surrounding areas. Additionally, trauma and intraoperative transplantation may also cause this spread [2,4].

The most common symptoms of SATMJ are pain, trismus, and swelling in the joint area. The rate of systemic symptoms was reported as 13.9%. The most common surgical intervention in SATMJ is arthrocentesis. In half of the cases where samples were taken for culture, there was no growth in culture. The most frequently occurring pathogen is *Staphylococcus aureus*, at a rate of 20.4% [1,5]. Since there was no growth in half of the culture, the diagnosis relies on the history, physical examination, laboratory findings, and imaging studies. Direct radiography, ultrasonography, CT, and MRI are commonly utilized as imaging modalities. It has been suggested that direct radiography may not detect the disease in its early stages, making CT and MRI more advantageous in this regard. Nevertheless, direct radiographs are most frequently employed in the diagnosis of SATMJ. While bone tissue destruction can be identified on direct radiographs, soft tissue assessment is not feasible through this method [1,2]. CT, on the other hand, allows for the detection of abscesses and alterations in surrounding soft tissues, in addition to bone tissue changes. An increase in joint space is a critical finding in SATMJ, and CT can also identify complications such as osteomyelitis and ankylosis [2,6,7]. MRI is highly sensitive for detecting joint fluid, evaluating joint surfaces, and identifying damage to surrounding soft tissues [2,7].

Early diagnosis and treatment are critical in preventing complications. Treatment begins with broad-spectrum empiric antibiotics. If culture can be obtained, treatment can be revised according to the results. While antibiotic therapy alone may be sufficient, surgical approaches that include drainage and resection may be needed depending on the condition of the case because inadequate treatment may result in systemic spread. Development of osteomyelitis, TMJ ankylosis, and trismus are among the possible complications [1,2]. A significant portion of TMJ ankylosis is thought to develop secondary to SATMJ that is not treated effectively [2]. In the study by Omiunu et al., 79.6% of cases in compilation recovered without complications [1].

Our case is one of the eldest patients in the literature. There was no predisposing factor in our case, and no etiological factor could be identified. The diagnosis was made by evaluating the history and performing a physical examination, blood tests, and imaging studies together. Another point that supports our diagnosis is the rapid response to antibiotic therapy. Since it was diagnosed at an early stage, there was no need for surgical intervention. For empirical antibiotic therapy, levofloxacin was chosen in addition to ampicillin-sulbactam due to its gram-negative activity and effective spread to cartilage and bone tissues [8]. With this treatment, a complication-free recovery was achieved.

## Conclusions

SATMJ should always be considered by clinicians in the differential diagnosis of TMJ diseases. Decreased oral intake due to loss of TMJ function in geriatric patients may predispose them to systemic spread. In our case, the absence of comorbid disease and the early initiation of effective treatment were the most important factors in the absence of sequelae and/or complications.
